# Hemoglobin concentration and blood shift during dry static apnea in elite breath hold divers

**DOI:** 10.3389/fphys.2024.1305171

**Published:** 2024-04-30

**Authors:** Thomas Kjeld, Thomas O. Krag, Anders Brenøe, Ann Merete Møller, Henrik Christian Arendrup, Jens Højberg, Dan Fuglø, Søren Hancke, Lars Poulsen Tolbod, Lars Christian Gormsen, John Vissing, Egon Godthaab Hansen

**Affiliations:** ^1^ Copenhagen Neuromuscular Center, Rigshospitalet, University of Copenhagen, Copenhagen, Denmark; ^2^ Department of Clinical Medicine, Panum Institute, University of Copenhagen, Copenhagen, Denmark; ^3^ Department of Anesthesiology, Herlev Hospital, University of Copenhagen, Copenhagen, Denmark; ^4^ Department of Cardiothoracic Anesthesiology, Rigshospitalet, University of Copenhagen, Copenhagen, Denmark; ^5^ Department of Nuclear Medicine, Herlev Hospital, University of Copenhagen, Copenhagen, Denmark; ^6^ Department of Nuclear Medicine and PET Centre, Aarhus University Hospital, Aarhus, Denmark

**Keywords:** cardiac PET/CT, cardiac MR (CMR), spleen ultrasound examination, dual x-ray absorptiometry (DXA), Bohr effect, free diving, myocardial mass, lower extremity blood volume

## Abstract

**Introduction:**

Elite breath-hold divers (BHD) enduring apneas of more than 5 min are characterized by tolerance to arterial blood oxygen levels of 4.3 kPa and low oxygen-consumption in their hearts and skeletal muscles, similar to adult seals. Adult seals possess an adaptive higher hemoglobin-concentration and Bohr effect than pups, and when sedated, adult seals demonstrate a blood shift from the spleen towards the brain, lungs, and heart during apnea. We hypothesized these observations to be similar in human BHD. Therefore, we measured hemoglobin- and 2,3-biphosphoglycerate-concentrations in BHD (*n* = 11) and matched controls (*n* = 11) at rest, while myocardial mass, spleen and lower extremity volumes were assessed at rest and during apnea in BHD.

**Methods and results:**

After 4 min of apnea, left ventricular myocardial mass (LVMM) determined by ^15^O-H_2_O-PET/CT (*n* = 6) and cardiac MRI (*n* = 6), was unaltered compared to rest. During maximum apnea (∼6 min), lower extremity volume assessed by DXA-scan revealed a ∼268 mL decrease, and spleen volume, assessed by ultrasonography, decreased ∼102 mL. Compared to age, BMI and VO_2_max matched controls (*n* = 11), BHD had similar spleen sizes and 2,3- biphosphoglycerate-concentrations, but higher total hemoglobin-concentrations.

**Conclusion:**

Our results indicate: 1) Apnea training in BHD may increase hemoglobin concentration as an oxygen conserving adaptation similar to adult diving mammals. 2) The blood shift during dry apnea in BHD is 162% more from the lower extremities than from the spleen. 3) In contrast to the previous theory of the blood shift demonstrated in sedated adult seals, blood shift is not towards the heart during dry apnea in humans.

*In memoriam Poul-Erik Paulev, 1935 - 2017*

## Introduction

Diving mammals like the adult Weddell seals (WS) possess an adaptive higher hemoglobin concentration and Bohr effect than pups and during simulated dives, sedated adult seals have been demonstrated to direct blood from the spleen to the heart, lungs and brain to meet metabolic requirements during dives, when partial pressures of oxygen (PaO_2_) decrease to 3.2 kPa (Zapol et al., 1979; Kjekshus et al., 1982; Kjekshus et al., 1982; Qvist et al., 1986). Elite breath-hold divers (BHD) are adapted to tolerate PaO_2_ to similar levels as demonstrated in the diving and foraging adult seals (Kjeld et al., 2018; Kjeld et al., 2021a; Kjeld et al., 2021c). However, in contrast to sedated seals, BHD decrease all internal cardiac chamber volumes ∼40% during apnea, whereas left ventricle wall thickness increases (Kjeld et al., 2021c), and the question is, whether the left ventricle wall thickness increase could be due to myocardial contraction or increased internal myocardial wall blood volume and hence a blood shift, as demonstrated in the adult seal? The adult hooded seals also have large spleens constituting 4% of their body volume and are capable of expanding the total circulating blood volume by up to 13% during dives as part of the mammalian diving response, and hereby increasing erythrocyte gas exchange capability (Hurford et al., 1996; Foster and Sheel, 2005; Kjeld et al., 2009; Kjeld et al., 2021a). These findings in diving mammals have led to studies of human apnea diving, which demonstrated splenic volume reductions of up to 170 mL and conclude that the spleen is an important reservoir of erythrocytes (Schagatay et al., 2001; Schagatay et al., 2005; Prommer et al., 2007; Schagatay et al., 2012; Ilardo et al., 2018; Bouten et al., 2019; Persson et al., 2023). However, considering that an average 70 kg man has a spleen size ∼200 mL, a blood volume of 5.5 L, of which 60% is in the musculoskeletal system and the lower extremities alone contain volumes of ∼ 2.2 L of blood (Karpeles and Huff, 1955; Adams and Albert, 1962), the question is whether lower extremity blood volume would be at least equally as important as the spleen to direct blood to the vital organs during apnea diving in humans?

As an adaptive response to chronic hypoxia, high-altitude species may also have higher concentrations of hemoglobin, changed standard half saturation pressures and a different Bohr effect than those of their lowland relatives (Vargas and Spielvogel, 2006) although this has been debated (Lenfant et al., 1968a; Winslow, 2007). The Bohr effect is a physiological phenomenon first described in 1904 by the Danish physiologist Christian Bohr: the binding affinity of the hemoglobin to oxygen is inversely related both to acidity and to the concentration of carbon dioxide (CO_2_) (Benner et al., 2023). Hence, the Bohr effect refers to the shift in the oxygen dissociation curve caused by changes in the concentration of CO_2_ or the pH of the environment. Since CO_2_ reacts with water to form carbonic acid, an increase in CO_2_ – like during apnea diving – results in a decrease in blood pH, resulting in release of oxygen by hemoglobin. Harbour seals also have a large fixed-acid Bohr coefficient at 37°C and increasing with temperature (Willford et al., 1990) – in contrast to humans (Boning et al., 1978). This relatively large value for the Bohr coefficient is similar to those reported for the Northern Elephant seals, Bladdernose seals, and Weddell Seals, and may facilitate oxygen off-loading as acidosis develops during a dive (Willford et al., 1990).

In competitive BHD, bouts of static and dynamic apnea increase plasma erythropoietin (Richardson et al., 2005; Kjeld et al., 2015). Hence, it may be that also the human elite BHD, that endure apneas of up to 11 mins and swimming more than 300 m, or going beyond 200 m in depth, all on a single breath of air (www.aida-international.org), would possess increased hemoglobin concentrations and oxygen offloading as an adaptation to (diving) hypoxia similar to diving mammals and high-altitude species.

Therefore, this study quantified 1) the oxygen binding properties of hemoglobin and 2,3 biphosphoglycerate (2,3-BPG) at rest in BHD as compared to matched controls, and 2) the left ventricle myocardial mass, spleen volume and lower extremity volume during apnea in elite BHD. BHD and controls were matched for age, body mass index (BMI), VO_2_max and spleen size (Chow et al., 2016). To ensure similar adaptations in the BHD in this study towards diving hypoxia as diving mammals, we required as inclusion criteria for BHD that they could hold their breath for a minimum of 5 min (Kjeld et al., 2018; Kjeld et al., 2021c). Also, to ensure similar adaptations as diving mammals towards diving hypoxia, we instructed BHD to pause aerobic training for 4 weeks before blood sampling (Bennett et al., 2001; Kjeld et al., 2018). Likewise, controls were instructed to pause anaerobic training for 4 weeks before blood sampling. Hence, we hypothesised that BHD as compared to controls would possess similar adaptations including binding properties of the hemoglobin to diving hypoxia as adult diving mammals, and also a blood shift from the spleen and lower extremities during apnea.

## Methods

22 healthy male, non-medicated, non-smoking participants were included in the study as approved by the Regional Ethics Committee of Copenhagen (H-1-2013-060). All clinical investigations have been conducted according to the principles expressed in the Declaration of Helsinki. Informed consent, written and orally, have been obtained from the participants.

Eleven participants were elite breath hold divers (BHD, age 44 ± 6 years), who were able to hold their breath for more than 5 min. For comparison we studied eleven judo athletes matched for morphometric variables (age, weight, body mass) and $\dot{V}$ O_2_max (Table 1; Figure 2) as described below. Judo athletes primarily train aerobic, and we have previously described judo athletes as controls in studies of BHD (Kjeld et al., 2018).

**TABLE 1:** Participants characteristics (*n* = 11 BHD & 11 controls).

	Divers	Controls	*p*
No. participants	11 males	11 males	NS
Age (years)	44 ± 2	37 ± 2	NS
Static breath hold personal best (seconds)	381 ± 15	N/A	NS
Height (cm)	189 ± 2	183 ± 1	0.015
Weight (kg)	83 ± 2	82 ± 2	NS
Body Mass Index (kg/m^2^)	23.3 ± 0.8	24.6 ± 0.6	NS
Spleen Volume/mL	230 ± 29	258 ± 30	NS
Maximal oxygen uptake (ml O_2_/(min*kg)	51.1 ± 2.7	56.8 ± 2.5	NS

Basic morphometric data. Values are mean ± Standard error of mean. *p*: level of significance. NS: non-significant. BHD, breath hold divers.

All BHD had ranked among national top 10, three of the participating free divers ranked among international top 10 and one was a 2016 outdoor free-diving World champion, one was a silver medalist at 2022 World Championships, and one was a World record holder.

All the matched controls were either judo or jiu-jitsu black belts, all were medalists at national championships, and all except one were active fighters.

This study included the following measurements of the BHD and matched controls (Figure 2; Table 2): Collection of blood samples for hemoglobin and 2,3-BPG at rest, a $\dot{V}$ O_2_ max test, ultrasonography of the spleen (US) at rest for both BHD and controls, whereas cardiac magnetic resonance imaging (cardiac MRI), positron emission tomography/computed tomography (PET-CT), ultrasonography of the spleen (US), and Dual Energy X-Ray Absorptiometry (DXA) measurements of the lower extremities during maximum apnea for BHD to ensure maximum cardiovascular response (Kjeld et al., 2021a).

**TABLE 2:** BHD (breath hold divers) participation in sub studies: Hb (hemoglobin), 2,3-BPG (2,3 biphosphoglycerate), CMRI (cardiac magnetic resonance imaging of left ventricle myocardial mass), PET-CT (positron emission tomography—computed tomography of left ventricle myocardial mass), US (ultrasound of the spleen volume), DXA (Dual Energy X-Ray Absorptiometry of the lower extremity volume).

BHD	Hb	2,3-BPG	CMRI	PET-CT	US	DXA
1	X	X	X	X		
2	X	X	X	X	X	X
3	X	X	X	X	X	X
4	X	X	X	X	X	X
5	X	X	X	X	X	X
6	X	X	X	X	X	X
7	X	X			X	X
8	X	X			X	X
9	X	X			X	X
10	X	X			X	X
11	X	X			X	X
Total	11	11	6	6	10	10

### 2,3-BPG measurements

Before taking blood samples from participants, BHD were carefully instructed to refrain from aerobic exercise for 4 weeks, and controls to refrain from anaerobic exercise for 4 weeks: we assumed that this respectively would decrease and increase levels of 2,3-BPG in BHD and controls (Lenfant et al., 1968b; Willford et al., 1990). However, these instructions did overall not change any habits of the subjects in the study.

Blood samples were taken from an antecubital vein on the first day of the study at rest in a supine position.

To prepare samples for 2,3-BPG measurement, 2 mL of venous blood in heparinized tubes was collected, placed immediately on ice, deproteinized with 0.6 M perchloric acid (Sigma-Aldrich, Saint Louis, MO, United States) to lyse red blood cells, and neutralized with 2.5 M potassium carbonate (Sigma-Aldrich, Saint Louis, MO, United States). The supernatant was kept for at least 60 min in an ice bath and centrifuged at 3,000 × g for 10 min. The supernatant was stored at 28°C, and 2,3-DPG levels were measured using the either Roche diagnostic kit (*n* = 3 BHD & *n* = 4 controls; no. 10148334001, Basel, Switzerland) or Cusabio Human 2,3- BPG (*n* = 8 BDH & *n* = 7 controls) ELISA Kit (Houston, TX, United States).

The Roche 2,3-DPG assay is based on enzymatic cleavage of 2,3-BPG, and oxidation of nicotinamide adenine dinucleotide recorded by spectrophotometry. The 2,3-BPG assays were performed in three batches and in the range of 0.02–0.15 μmol (*n* = 3 BHD and 4 controls). Concentration of 2,3-BPG was calculated according to the procedure proposed by the manufacturer. The 2,3-BPG levels were normalized to the corresponding hematocrit value from the same sample. Since the concentration of 2,3-BPG rapidly decreases during storage (Hamasaki and Yamamoto, 2000), the procedure for determining the 2,3-BPG level was performed immediately after taking the blood samples. Determination of 2,3-BPG level was carried out in duplicate on each sample. The reliability of 2,3-BPG measurement was evaluated based on the coefficient of variation (CV) using the test–retest method (Atkinson and Nevill, 1998). CV for 2,3-BPG was between 0.30 and 0.76%, which indicates that these measurements are characterized by a high degree of reliability.

Production of the Roche assay had ceased when the remaining participants participated. Hence, for the remaining participants (*n* = 8 BHD and 7 controls), 100 µL plasma was analyzed in triplicate for each participant using a 2,3-BPG ELISA kit manufactured by Cusabio (Houston, TX). Briefly, analysis of samples were done according to manufacturer’s instructions. All incubation was done at 37 degrees and absorbance was read at 450 nm, using wavelength correction by subtracting absorbance reading at 570 nm from absorbance read at 450 nm (Thermo Scientific Multiskan Go, Waltham, MA). Concentrations of 2,3-BPG were determined, using the average of the triplicate absorbances for each sample, from a four-parameter logistic curve of the absorbance of the standard at various concentrations.

The blood sample analyzes of two controls and one BHD failed and could not be repeated.

Hb was determined on an ABL 90 Flex (Radiometer, Bronshoj, Denmark). Concentration of 2,3-BPG in mM was calculated per mM Hb, Analysis of samples from two controls and one BHD failed and could not be repeated.

### $\dot{V}$ O_2_ max

After blood sampling, participants completed a standardized warm-up followed by an incremental cycle test (Lode®) starting at a workload of 50 W and increasing 50 W every minute until exhaustion (Astrand and Ryhming, 1954). The highest recorded 30 s average oxygen uptake ( $\dot{V}$ O_2_) during the test was defined as $\dot{V}$ O_2_max. For recognition of true $\dot{V}$ O_2_max, three of five criteria had to be met: individual perception of exhaustion, respiratory exchange ratio >1.15, plateau of $\dot{V}$ O_2_ curve, heart rate approaching age-predicted maximum and inability to maintain a pedaling frequency above 70 rpm (Table 1).

### Cardiac magnetic resonance imaging: image acquisition

On a separate day, the 6 participants with the longest breath holds refrained from physical exercise and consumption of alcohol or caffeine for 24 h before the following was performed: Imaging was performed in a 1.5 T cardiac MRI imaging system (Achieva, Philips Medical System, The Netherlands). Subjects warmed up by holding their breath three consecutive times with individual duration to maximize the diving response including the blood shift (Kjeld et al., 2009; Kjeld et al., 2021c). Cine images were acquired at 1) rest during a short (<20 s) apnea at end-expiration with open pharynx, 2) after 4 min of dry static apnea after glossopharyngeal insufflation (Seccombe et al., 2006). Images were collected shortly before end of apnea before breathing, and participants were instructed to stay as calm as possible during imaging to avoid imaging artefacts. Left ventricular myocardial mass was collected in the transversal and double-oblique short axis stacks with 8 mm thick slices and 25% gap. Cine imaging was performed with retrospectively ECG-gated steady-state free precession sequences (SSFP) reconstructed to 25 phases covering the entire cardiac cycle using the following settings: TR/TE 3.3/1.6 ms, flip angle 60°; and spatial resolution 1.3 × 1.3 × 8 mm^3^ as previously described (Kjeld et al., 2021c).

### Cardiac magnetic resonance imaging: image analysis

**FIGURE 1:**
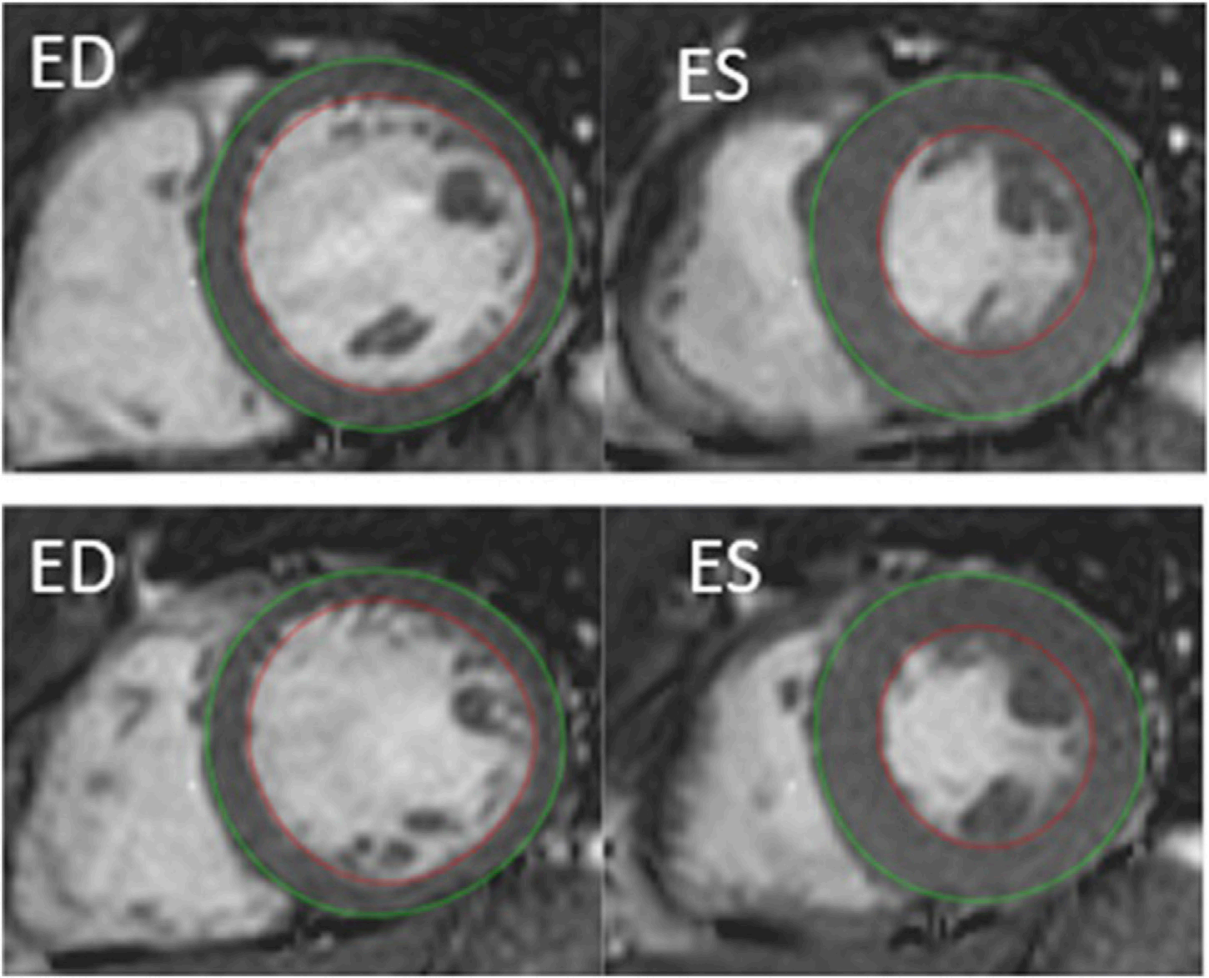
Example of cardiac MRI determined myocardial mass by semi-automatic segmentation of the left ventricle, short-axis view. Delineation of endocardial contour in red and epicardial contour in green. ED, end diastolic. ES, end systolic. Top: rest. Bottom: after 4 min of apnea.

Left ventricular myocardial mass was determined in end-diastole (ED) and end-systole (ES, Figure 1). Data were analyzed by a level three nuclear physiologist and a level one Cardiac MRi cardiologist using dedicated software (Segment ^®^ version 2.1, Lund, Sweden). Image artefacts were carefully avoided especially during involuntary breathing movements (Heiberg et al., 2010) and using a fitting algorithm in the Segment software ^®^ as previously described (Bidhult et al., 2016; Kjeld et al., 2021c).

**FIGURE 2:**
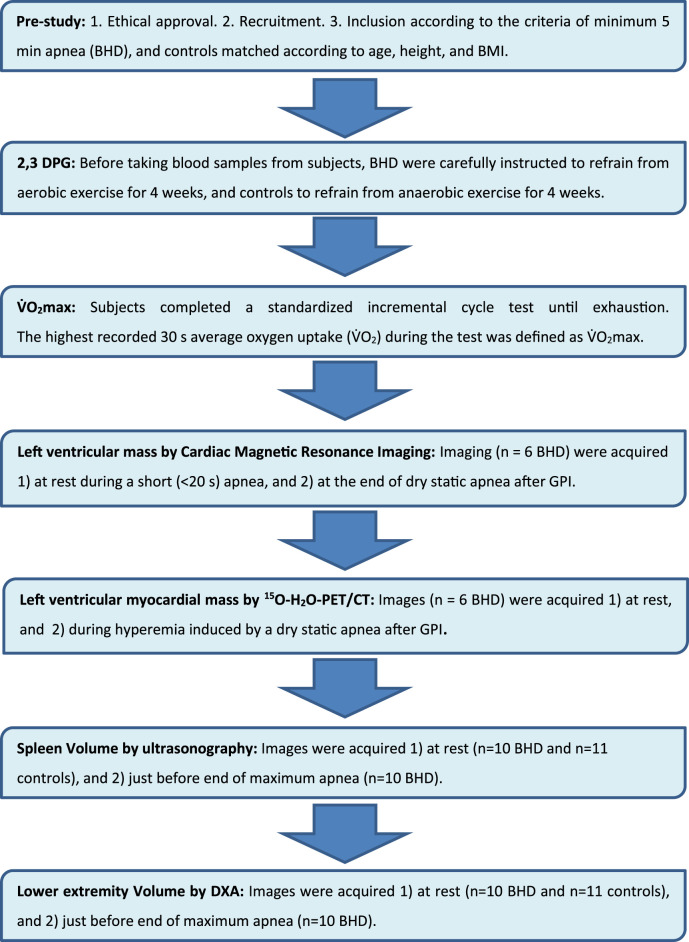
Flow chart.

### ^15^O-H_2_O-PET/CT determined left ventricular myocardial mass: imaging protocol and image reconstruction

On a separate day, the same six participants who had cardiac MRI were recruited for an additional ^15^O-H_2_O-PET/CT study. The participants in the ^15^O-H_2_O-PET/CT sub-study were required to hold their breath for 5 min while lying in the PET/CT scanner in the supine position with arms raised above the head. They were instructed to refrain from intake of chocolate, to refrain from strenuous physical exercise for 1 day and to be fasting for at least 6 h before the study.

^15^O-H_2_O-PET/CT data were obtained in list mode on a GE Discovery MI Digital Ready PET/CT system (GE, Milwaukee, WI, United States) as described previously (Kjeld., et al., 2021a). In short, the participants were scanned using ^15^O-H_2_O cardiac perfusion PET/CT 1) at rest, 2) during hyperemia induced by a dry static apnea after glossopharyngeal insufflation (Seccombe et al., 2006) and after a warm-up of three individual consecutive apneas to maximize the diving response including the blood shift (Kjeld et al., 2009), and 3) in the recovery phase 4 min after the apnea.

### ^15^O-H_2_O-PET/CT determined left ventricular myocardial mass: image analysis

**FIGURE 3:**
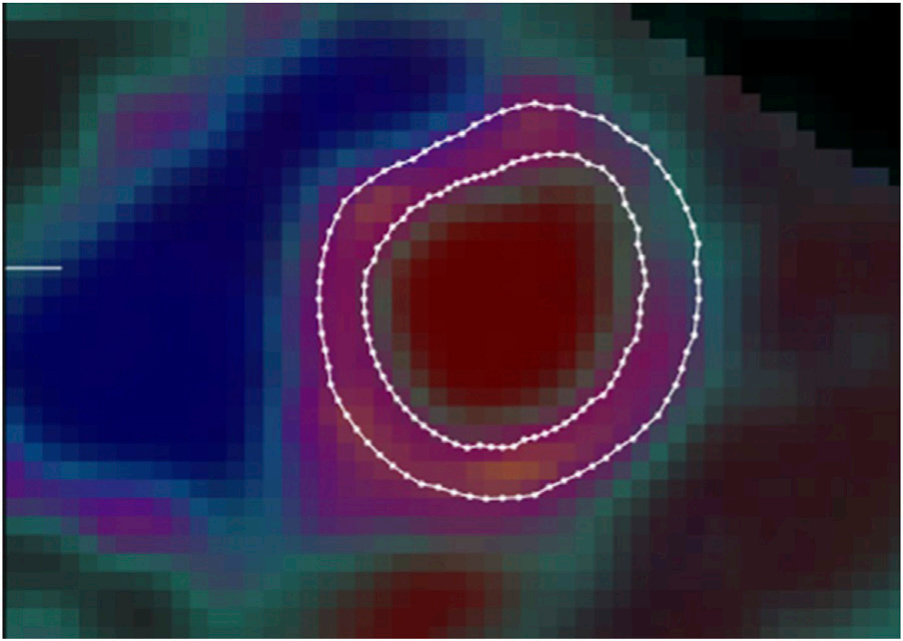
Example of left ventricular myocardial mass image as determined by ^15^O-H_2_O-PET/CT using semi-automatic segmentation of the left ventricular wall on parametric images of perfusable tissue fraction. Borders of endocardium (inner circle) and pericardium (outer circle) defined by delineated lines.

Left ventricular mass (LVM) was quantified by semi-automatic segmentation of the LV wall on parametric images of perfusable tissue fraction (PTF) as previously described (Sorensen et al., 2021). In short, parametric images were obtained by kinetic analysis of the dynamic ^15^O-H_2_O-PET/CT scan using a 1-tissue compartment model with image derived input from cluster analysis (Harms et al., 2011)In the model, PTF accounts for partial volume effects on the difference between myocardial blood flow estimated from ^15^O-H_2_O wash-in and wash-out. The PTF parameter is more robust for segmentation compared to myocardial blood flow since it is less affected by perfusion defects and segmentation has been shown to be highly reproducible (Sorensen et al., 2021) (Figure 3).

### Spleen volume

On a separate day, the participants had ultrasonography (US) of the spleen using an Esaote ^®^ scanner (Mylab, Omega, Genova, Italy, 2017) with a 3.5/5-MHz convex transducer probe. Spleen metrics were assessed by using defined standard algorithms according to Koga T. (1979). With the participants in the supine position after approximately 15 minutes of rest, the examination started in the posterior axillary line in the approximate area of the 10th rib through an intercostal space to identify the longitudinal view of the spleen with the hilus. In this position, maximum length, and width of the spleen was measured on a frozen high resolution ultrasound image.

During breath holds (BHD only), the diaphragm changes the position of the spleen, and to diminish measuring artefacts, we decided only to measure the spleen in transverse axis. Hence, the maximum length and the maximum width of the spleen was determined, and according to the formula by Koga T. (1979), the spleen volume calculates to

$$V=7.53\;x\;0,8\;x\;\left(\text{length }x\text{ width}\right)\;–\;77,56$$

The above was performed minimum three times in all participants at rest.

**FIGURE 4:**
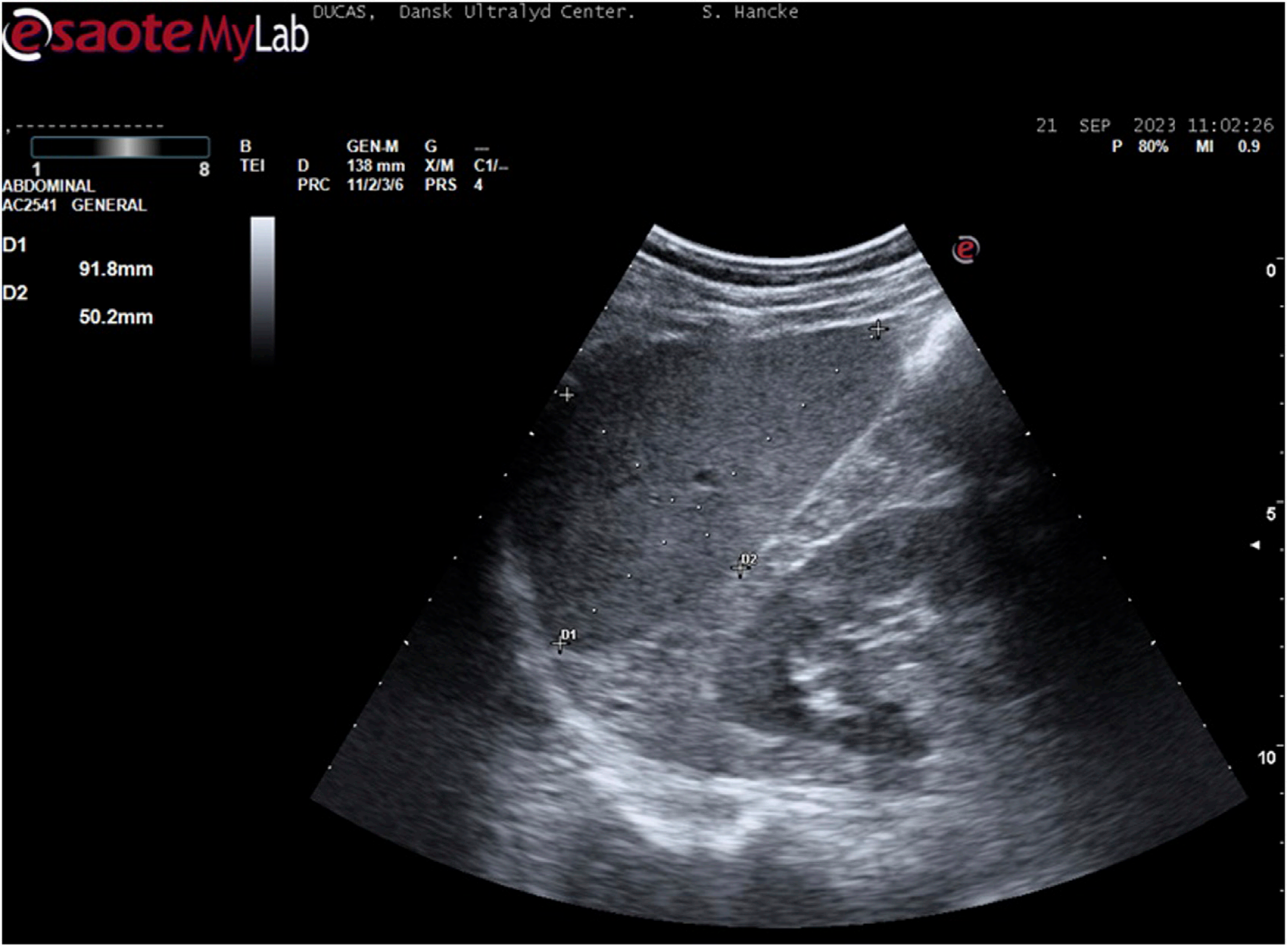
Example of spleen measurement (participant 6): With the participant in the supine position, the examination started in the posterior axillary line in the approximate area of the 10th rib through an intercostal space to identify the longitudinal view of the spleen with the hilus. In this position, maximum length and width were measured.

After a warm-up of three individual consecutive submaximal apneas with short pauses (minimum 4 and maximum 7 minutes pause) in between (Kjeld et al., 2014), BHD performed a following maximum apnea to maximize the diving response including the blood shift (Kjeld et al., 2009). Hence, the above-described measurements at rest were repeated after 4 min of maximum apnea and up to 4 additional measurements were made until just before termination of apnea in the BHD (Figure 4).

### Lower extremity DXA scan

**FIGURE 5:**
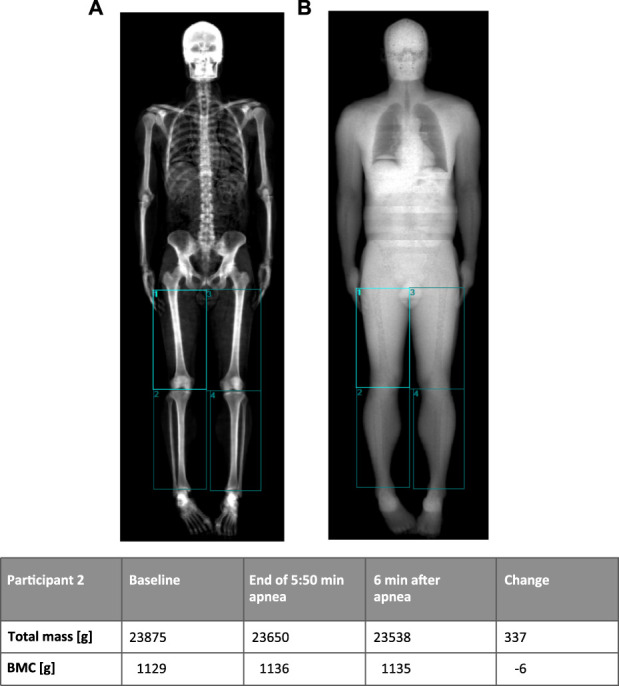
Example of DXA-scan (participant 2) with manually drawn regions in the lower extremities using bone tissues **(A)** and soft tissue **(B)** images to place regions. The table shows changes in mass of the lower extremities (sum of the four regions) before and after 5 min 50 s breath hold. Note that the bone mineral content (BMC) remains unchanged, indicating minimal movement of the participant (A image).

On a separate day, three consecutive whole body scans were performed, and data included total body weight bone mineral content, fat free mass and lean mass. The first and the third scans were routine whole-body scans at rest. After the first scan, the BHD then performed three sub-maximal apneas with short pauses (minimum 4 and maximum 7 minutes) in between to warm up (Kjeld et al., 2014), and BHD were instructed to do similar a warm-up as described above during the ultrasonography study. The second scan was following a short pause after the warm-up and during maximum apnea, of which the last part of the scan including the legs, was timed to be initiated 3 minutes and 30 seconds after initiation of apnea and finished after 4 minutes of apnea to ensure a maximum diving response (Kjeld et al., 2021a). There was no repositioning between scans except for movement of one arm in one case, where the diver needed to adjust the nose clip. The Table 3 shows changes in mass in tissue compartments of the lower extremities. Note that the total mass (including blood pool) changes whereas the bone mineral content remains unchanged indicating minimal movement of the participant. The change in mass of the lower extremities is attributed to a change in blood pool as other tissue compartments are not as volatile to change. All scans were performed on a GE Lunar iDXA scanner (GE Medical Systems, Madison, WI). Regions of interest over the legs were analyzed according to the method used by (Gjorup et al. (2017) (Figure 5).

**TABLE 3:** ^15^O-H_2_O-PET/CT and cardiac MRi left ventricle myocardial mass (LVMM), ultrasonography assessed spleen volume and DXA lower extremities total mass and bone mineral content (BMC) of 11 breath hold divers.

	Rest	Apnea
^15^O-H_2_O-PET/CT assessed LVMM/g	149 ± 11	146 ± 12
Cardiac MRi assessed LVMM/g	116 ± 6	112 ± 5
Ultrasonography assessed spleen volume/mL	230 ± 29	128 ± 21 (*1, *2)
DXA assessed lower extremities total mass (g)	23,792 ± 1,084	23,546 ± 1,077 (*1, *2)
DXA assessed lower extremities BMC (g)	982 ± 37	984 ± 38

Basic morphometric data. Values are mean ± Standard error of mean. *1: *p* < 0.001 compared to rest. *2: *n* = 10: 1 subject moved abroad during the study.

### Statistical analysis

To test for imbalances in the variables in our study with a limited number of participants, we performed a power calculation: The sample size of 6 participants in the ^15^O-H_2_O-PET/CT sub-study was calculated for the primary outcome (myocardial blood flow) of the study previously published by our group (Kjeld et al., 2021c). A power calculation was performed *post hoc* but based on the test-retest standard deviation of 19 g determined by Sorensen et al. (Sorensen et al., 2021). The current analysis had a power of 80% to detect a difference of at least 55 g. A *p*-value <0.05 was considered statistically significant. This was also the basis for the sample size of the sub study of cardiac MRI, and also the other sub-studies as the latter had more participants.

Variables are presented as mean ± standard error of the mean (SEM). Data were analyzed by Sigma-Plot ^®^ using one-way repeated measures ANOVA. Holm-Sidaks method posthoc was used to evaluate differences between the collected data during rest, apnea, and recovery. A *p*-value <0.05 was considered statistically significant.

## Results

BHD were higher (189 ± 2 cm) than controls (183 ± 1 cm, *p* = 0.015), but their spleen volumes were similar (Tables 1, 3).

**FIGURE 6:**
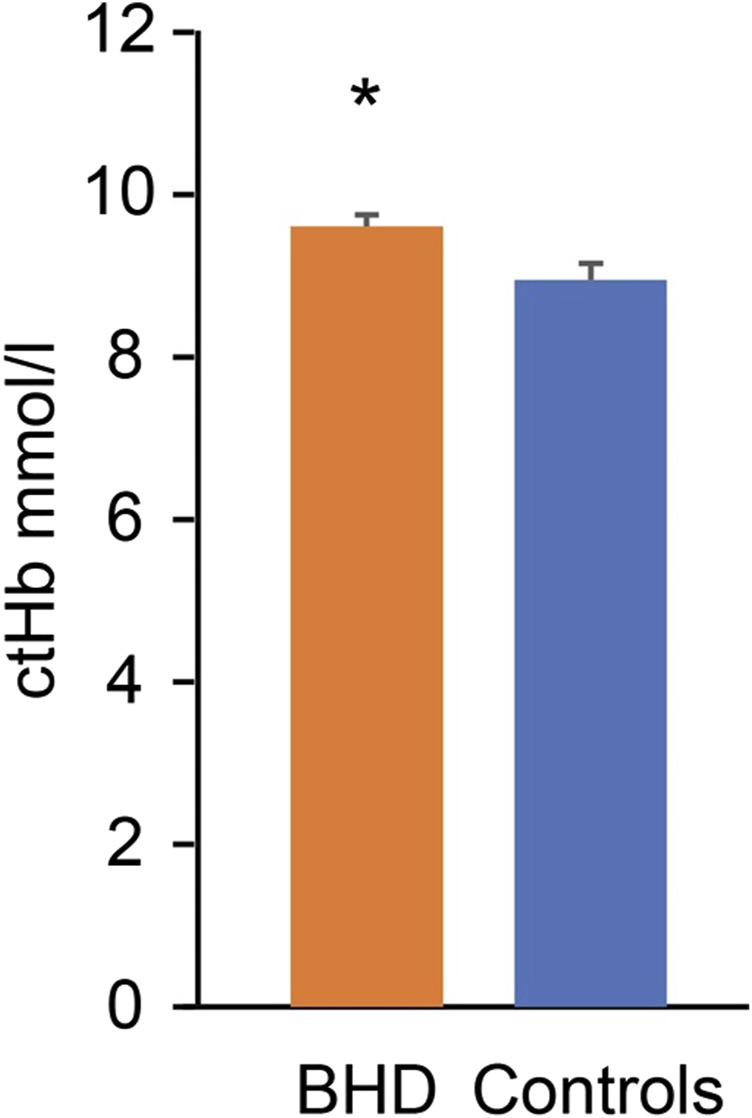
Hemoglobin concentration (ctHb) in breath hold divers (BHD, 9.7 ± 0.1 mmol/L) as compared to controls (9.1 ± 0.2 mmol/L). *: *p* = 0.038. Error bars: Standard error of the mean.

Compared to the matched controls, the BHD had similar content of 2,3-BPG (BHD 0.138 ± 0.025 vs. controls 0.119 ± 0.009 mM, *p* = 0.534), but they had a higher concentration of hemoglobin (*p* = 0.038; Figure 6).

Subgroups of BHD who underwent cardiac PET-CT, cardiac MRI, ultrasonography of the spleen and DXA, respectively, were not significantly different when comparing their age, height, weight, BMI, $\dot{V}$ O_2_max and apnea duration (Table 1).

After 2 and 4 min of apnea, ^15^O-H_2_O-PET/CT determined left ventricular myocardial mass (149 ± 11 g) was unaltered as compared to rest (146 ± 12 g; n = 6). Images of one participant were of poor quality and could not be repeated. Therefore, results from only 5 participants could be obtained from this protocol.

After 4 min of apnea, cardiac MRI determined left ventricular myocardial mass (116 ± 6 g) was unaltered as compared to rest (112 ± 5 g; *n* = 6).

Spleen volumes at rest were not different between controls (n = 11) and BHD (*n* = 10: one participant moved abroad during the study; Table 1). During maximum apnea (370 ± 67 s) the spleen volumes of BHD decreased from 230 ± 29 to 128 ± 21 mL (*p* < 0.001; Table 3).

Whole-body DXA-scan of BHD revealed a total body weight of 81.2 ± 2.8 kg (*n* = 10: one participant moved abroad during the study).

During maximum apnea (343 ± 9 s) the lower extremity mass decreased from 23,792 ± 1,084 g at rest to 23,741 ± 1,081 g (*p* = 0.2) after 4 min of apnea and decreased further at the end of apnea to 23,546 ± 1,077 g (*p* < 0.001; Table 3). The bone mineral content in the regions remained unchanged indicating minimal movement of the participants (rest 982 ± 37 g, during apnea 984 ± 38 g, after apnea 984 ± 38 g).

## Discussion

The main and novel findings of our study are as follows: After a warm-up of three consecutive apneas, BHD have 1) unaltered left ventricle myocardial mass after 2–4 min of apnea, 2) a decreased spleen volume by ∼102 mL during maximum apnea, and 3) a decreased lower extremity weight by 268 g after maximum apnea, indicating 268 mL less blood volume. These results indicate that the blood shift during apnea in elite BHD– at least partly– comes from the lower extremities and from the spleen, but the blood shift is not towards the heart in contrast to observations in sedated adult diving mammals. The present study also demonstrated that BHD have a higher concentration of hemoglobin, but similar 2,3-BPG levels as compared to controls matched for BMI, age, spleen volume and $\dot{V}$ O_2_max.

Our results demonstrate for the first time in BHD, that the blood shift during maximum apnea after glossopharyngeal insufflation is only partly similar to the blood shift found in other diving mammals. Also, we suggest that the higher hemoglobin in BHD as compared to controls is an adaptation to sustain hypoxia during dives similar to adult diving mammals, that have higher hemoglobin mass and larger spleens relative to terrestrial mammals.

Relatively large Bohr effects are observed in adult Harbour seals, killer whales (Lenfant et al., 1968a) and Northern Elephant seals (Willford et al., 1990), especially in tissues with low oxygen content, for example, their skeletal muscles. Previously we have demonstrated a mitochondrial adaptation to hypoxia in the skeletal muscles of BHD similar to the adult northern elephant seal (Kjeld et al., 2018). As adult seals have a higher mitochondrial respiratory capacity and higher muscular myoglobin concentration than juvenile seals, which are not yet fully matured and adapted to long dives (Kanatous et al., 2008; Chicco et al., 2014), the above suggests that adaptations leading to increased skeletal muscle oxygen storage and diffusion capacity is associated with improved diving performance. Hence, a similar Bohr effect as demonstrated in diving mammals could be expected in the BHD in our study could be expected. However, Bohr effects in BHD and controls in our study were similar, whereas hemoglobin concentrations differed: Accordingly, no high altitude training studies have demonstrated permanent changes in Bohr effects or 2,3-BPG content nor permanently in hemoglobin concentration after returning from high altitude (Gore et al., 2006; Millet et al., 2010; Faiss et al., 2013): For example, Ploszczyca K. et al. demonstrated a decrease in 2,3-BPG just after exercise, likely due to changes in pH and CO_2_ (Ploszczyca et al., 2021) and Elia et al., demonstrated higher hemoglobin levels, but lower mean cell volume and similar hematocrits in BHD compared to BMI matched controls, but not matched for $\dot{V}$ O_2_max (Elia et al., 2019). Our previous studies included BHD and controls with comparable levels of hemoglobin (Kjeld et al., 2018; Kjeld et al., 2021a; Kjeld et al., 2021c), but BHD in the previous studies were not instructed as in the present study to refrain from aerobic exercise 4 weeks before blood samples were taken and similarly for controls to refrain from anaerobic exercise for 4 weeks before blood samples were taken.

Hence, our results indicate that apnea training in BHD as compared to $\dot{V}$ O_2_max matched controls may increase hemoglobin concentration, however within normal reference values, but not 2,3-BPG.

Adult seals have increased hematocrits as compared to pups (Thomas and Ono, 2015). Also, South American natives living in high altitude with chronic hypoxic exposure have higher hemoglobin concentrations, than those of their lowland relatives (Boning et al., 1978; Vargas and Spielvogel, 2006; Storz and Moriyama, 2008; Böning, 2019). These natives are also demonstrated to decrease lactate concentrations during maximum exercise (Ge et al., 1994; Hochachka et al., 2002), similar to elite BHD during max apneas (Kjeld et al., 2021a; Kjeld et al., 2021a; Kjeld et al., 2021b; Kjeld et al., 2021c; Kjeld et al., 2021c). The increased hematocrits in high altitude natives due to hypobaric living are of interest in lowland living endurance athletes (Faiss et al., 2013), and hence hypobaric training has had focus to cause increased hematocrits in these athletes (Zelenkova et al., 2019), however, effects remain unproven (Hauser et al., 2016; Hauser et al., 2017). Accordingly, all the participants in our study had hemoglobin levels within normal range Hence, the explanation for an adequate oxygen supply to tissues under intermittent hypoxic conditions in BHD are not solely the oxygen carrier capacity in the blood, but also in the tissues, including the skeletal muscles similar to diving mammals, as demonstrated previously (Kjeld et al., 2018): relative to comparable terrestrial mammals– the skeletal muscles of seals and sea lions have higher mitochondrial volume densities and correspondingly higher citrate synthase activity, higher beta-hydroxyacyl CoA dehydrogenase activity and thus a higher capacity for fatty acid catabolism for aerobic ATP production (Kanatous et al., 2002). These adaptations may be speculated to result in higher mitochondrial respiratory capacity, but strikingly, the oxidative phosphorylation capacity of the northern elephant seal muscle is generally lower than in non-diving humans (Chicco et al., 2014). Interestingly, the skeletal muscles of BHD, compared to matched aerobic athletes, are also characterized by lower mitochondrial oxygen consumption both during low leak and high electron transfer system respiration indicating slow muscle oxygen consumption (Kjeld et al., 2018).

The movement pattern of the Northern Elephant Seal is impressive: they travel up to 13.000 miles per year, but swimming is slow: 1-2 m per second with less than 1 swimming stroke per second (Adachi et al., 2014). This swimming pattern is similar to elite BHD during their competitions and anaerobic exercise. Aerobic exercise increases blood flow 10-fold in the lower extremities of untrained individuals and up to 16 times in endurance cyclist and swimmers (∼ 4,500 mL/min) (Walther et al., 2008). In comparison, the blood shift during apnea mediated by the contraction of the human spleen is ∼ 102 mL in the present study. The human spleen contains 2-3% of total erythrocytes mass or 20–40 mL of erythrocytes (Wadenvik and Kutti, 1988), of which– if all can be assumed to be released– would increase blood volume in our study by ∼ 256 mL. In our study we demonstrated that blood volume expelled from the lower extremities is 268 mL during dry apneas in BHD, and hence the legs are reservoir of blood volume of at least equal importance as compared to the spleen in humans (Schagatay et al., 2005; Schagatay et al., 2012; Ilardo et al., 2018): Diving mammals have short limbs with high content of myoglobin (Wright and Davis, 2006) in contrast to elite BHD (Kjeld et al., 2018), and the size of for example, the spleen of the hooded seals (Cystophora cristata, weighing ∼ 250 kg) is up to 4% of the total body mass, and expels ∼ 13% of the total blood volume during dives (Cabanac et al., 1997). Accordingly, the physiological characteristics observed in adult diving mammals during apnea and underwater swimming are therefore not similar to terrestrial mammals during exercise: During aerobic exercise, terrestrial mammals increase ventilation, heart rate, cardiac output, and peripheral vasodilation, and the latter increases skeletal muscle perfusion (Wagner, 1991). By contrast, adult diving mammals during apnea and underwater swimming are characterized by bradycardia, decreased cardiac output and peripheral vasoconstriction including contraction of the spleen, which collectively is known as the dive response, and this physiological adaptive phenomenon has so far been interpretated as similar in elite BHD (Kjeld et al., 2009; Kjeld et al., 2014; Kjeld et al., 2015; Kjeld et al., 2018; Kjeld et al., 2021a; Kjeld et al., 2021c): A blood shift has been measured with ultrasonography and by injecting microspheres in anesthetized adult seals, placed with their heads downwards immersed in ice water, and it included centralization of blood to the brain, heart and lungs, and hence all peripheral tissue, including muscles and splanchnic organs, have reduced convective oxygen delivery resulting from both hypoxic hypoxia and ischemic hypoxia (Zapol et al., 1979; Hurford et al., 1996). However, studies of restrained and sedated seals during apnea, may demonstrate the physiological consequence of the sedation rather than the dive response, as the sedation depresses the cardiovascular system including the myocardial regional oxygen supply (Kanto, 1988), and cannot be compared to the foraging animal with an increased metabolic demand during their dives with concomitant hypoxia. Yet, our previous study demonstrated that left ventricle wall thickness increases during apnea in BHD, but that all internal cardiac chamber volumes decreases ∼ 40% (Kjeld et al., 2021c). As the results of the present study indicates unaltered myocardial mass during apnea in BHD as compared to rest, we suggest that there is not a blood shift towards the heart in BHD as observed in sedated adult seals during apnea. Together these results indicates a remodeling of the myocardium during apnea and we suggest the remodeling to be similar as observed in a cardiac MRI animal study of cardiac arrest and as discussed previously (Berg et al., 2005; Kjeld et al., 2021c).

## Conclusion

Our results indicate the following: 1) elite BHD compared to matched controls are adapted with increased hemoglobin concentrations to sustain extreme hypoxia, and 2) the blood shift in elite BHD during maximum apnea after glossopharyngeal insufflation is– at least partly– direction of blood from the lower extremities and with a smaller volume from the spleen, but 3) in contrast to previous studies of sedated diving mammals during apnea, our study of elite BHD do not demonstrate a blood shift towards the heart.

## Perspectives

In our study BHD endured maximum apneas of 370 s on average. Previously we have demonstrated similar duration of apnea in BHD and similar tolerance for low PaO_2_ during apnea as compared to diving mammals (Kjeld et al., 2021c). The BHD in the present study had unaltered left ventricular myocardial mass volumes after 2-4 min of apnea but decreased spleen volumes to 102 mL during maximum apnea, whereas extremities released 268 mL of blood, or ∼162% more than the spleen. Mijacika et al. demonstrated a large decrease in pulmonary blood volume after glossopharyngeal insufflation following 4 min of apnea, and hence, we suggest that blood released from lungs, spleen and lower extremities are directed towards the abdomen. Accordingly, the intestine, the kidneys and the liver are highly sensitive to hypoxia in humans (Ebert, 2006; Fu et al., 2016; Mijacika et al., 2017a; Mijacika et al., 2017b; Singhal and Shah, 2020), whereas the heart, lungs and skeletal muscles seem resistant to hypoxic injury during maximum apnea in elite BHD (Kjeld et al., 2015; Kjeld et al., 2018; Kjeld et al., 2021c). Future studies may reveal whether blood is directed from the extremities towards the hypoxia sensitive abdominal organs as an oxygen conserving mechanism, which could explain the evolutionary development of short flat peripheral extremities in diving mammals (Goldbogen, 2018).

## Limitations

The study population is small, because of the limitations in the inclusion criteria of minimum 5 min of apnea in different stressful environments. This limited the number of participants in the sub studies. In addition, measuring 2,3-BPG and hemoglobin concentrations compared to a control group instead of prospective measurement in BHD, is a limitation in our study. However, because we found no changes in the study of LVMM, and significant results in the rest of the sub studies, we assume this is of minor importance, although measurements of LVMM were after 4 min of apnea and not at end of maximum apnea (∼6 min).

The study did not include measurements of the atrial and right ventricle mass as this was not possible due the thin walls of these structures. However, our previous studies indicated no changes in pro-atrial-natriuretic factor during maximum apnea in competitive BHD indicating no stretching of atrial muscle, and as the right ventricle is much less muscular than the left (Rodrigues et al., 2007), we assume that changes in these chambers may be of lesser importance (Kjeld et al., 2015; Kjeld et al., 2021c). Measurements were all done during dry apneas after glossopharyngeal insufflation, which limits venous return (Eichinger et al., 2010). As submersion during diving may cause 500-700 mL increase in circulating volume (Weston et al., 1987; Weenink and Wingelaar, 2021) future studies may reveal the most important reservoirs of blood and volume changes during human apnea diving.

## Data Availability

The data that support the findings of this study are saved encrypted at hospital servers, but restrictions apply to the availability of these data, which were used under license for the current study, and so data are not publicly available. Data are however available from the authors upon reasonable request and with permission from Region Hovedstaden, Herlev Hospital, Skejby Hospital and Rigshospitalet.
