# Correction: Hemoglobin concentration and blood shift during dry static apnea in elite breath hold divers

**DOI:** 10.3389/fphys.2026.1933950

**Published:** 2026-07-22

**Authors:** Frontiers Production Office

**Affiliations:** Frontiers Media SA, Lausanne, Switzerland

**Keywords:** cardiac PET/CT, cardiac MR (CMR), spleen ultrasound examination, dual x-ray absorptiometry (DXA), Bohr effect, free diving, myocardial mass, lower extremity blood volume

In the published article there was an error in the reporting of one of the percentages. It was given as 20% when it should have been 60%.

A correction has been made to the section **Introduction,** *paragraph 1*:

“However, considering that an average 70 kg man has a spleen size ∼200 mL, a blood volume of 5.5 L, of which 60% is in the musculoskeletal system and the lower extremities alone contain volumes of ∼ 2.2 L of blood (Karpeles and Huff, 1955; Adams and Albert, 1962), the question is whether lower extremity blood volume would be at least equally as important as the spleen to direct blood to the vital organs during apnea diving in humans?”

The original version of this article has been updated.

